# Association of monocyte HLA-DR expression over time with secondary infection in critically ill children: a prospective observational study

**DOI:** 10.1007/s00431-021-04313-7

**Published:** 2021-11-10

**Authors:** Nienke N. Hagedoorn, Pinar Kolukirik, Nicole M. A. Nagtzaam, Daan Nieboer, Sascha Verbruggen, Koen F. Joosten, Henriette Moll, Gertjan Driessen, Willem A. Dik, Clementien Vermont

**Affiliations:** 1 Department of General Pediatrics, Erasmus MC Sophia, University Medical Center Rotterdam, Rotterdam, The Netherlands; 2 Pediatric Infectious Diseases & Immunology, Erasmus MC Sophia, University Medical Center Rotterdam, Rotterdam, The Netherlands; 3 Department of Immunology, Laboratory Medical Immunology, Erasmus MC, University Medical Center Rotterdam, Rotterdam, The Netherlands; 4 Department of Public Health, Erasmus University Medical Center, Rotterdam, The Netherlands; 5 Pediatric Intensive Care, Erasmus MC-Sophia, University Medical Center Rotterdam, Rotterdam, The Netherlands; 6 Department of Pediatrics, Maastricht University Medical Center, Maastricht, The Netherlands

**Keywords:** Immunosuppression, Secondary infection, Critical care, Pediatrics

## Abstract

An impaired immune response could play a role in the acquisition of secondary infections in critically ill children. Human leukocyte antigen-DR expression on monocytes (mHLA-DR) has been proposed as marker to detect immunosuppression, but its potential to predict secondary infections in critically ill children is unclear. We aimed to assess the association between mHLA-DR expression at several timepoints and the change of mHLA-DR expression over time with the acquisition of secondary infections in critically ill children. In this prospective observational study, children < 18 years with fever and/or suspected infection (community-acquired or hospital-acquired) were included at a paediatric intensive care unit in the Netherlands. mHLA-DR expression was determined by flow cytometry on day 1, day 2–3 and day 4–7. The association between delta-mHLA-DR expression (difference between last and first measurement) and secondary infection was assessed by multivariable regression analysis, adjusted for age and Paediatric Logistic Organ Dysfunction-2 score. We included 104 patients at the PICU (median age 1.2 years [IQR 0.3–4.2]), of whom 28 patients (27%) developed a secondary infection. Compared to 93 healthy controls, mHLA-DR expression of critically ill children was significantly lower at all timepoints. mHLA-DR expression did not differ at any of the time points between patients with and without secondary infection. In addition, delta-mHLA-DR expression was not associated with secondary infection (aOR 1.00 [95% CI 0.96–1.04]).

*Conclusions*: Our results confirm that infectious critically ill children have significantly lower mHLA-DR expression than controls. mHLA-DR expression was not associated with the acquisition of secondary infections.

**Table:** 

**What is Known:** *• An impaired immune response, estimated by mHLA-DR expression, could play an essential role in the acquisition of secondary infections in critically ill children.* *• In critically ill children, large studies on the association of mHLA-DR expression with secondary infections are scarce.*	
**What is New:** *• Our study confirms that critically ill children have lower mHLA-DR expression than healthy controls.* *• mHLA-DR expression and change in mHLA-DR was not associated with the acquisition of secondary infection.*

**Supplementary information:**

The online version contains supplementary material available at 10.1007/s00431-021-04313-7.

## Introduction

Critically ill patients are at risk for a prolonged period of immunosuppression, potentially leading to an increased risk of secondary infections [1–4]. Secondary infections, acquisition of an infection during hospital admission, occur in 11 to 19% of paediatric intensive care unit (PICU) admissions [5–7], causing prolonged hospital stay, morbidity, mortality and costs [8].

Immunosuppression has been established in critically ill patients following trauma, surgery or stroke [9–14], but most studies have focused on adult patients with sepsis and septic shock [3, 15–22].

Monocytic human leukocyte antigen-DR (mHLA-DR) expression has been shown a reliable biomarker in critically ill patients to estimate immunosuppression. In adults, a prolonged decrease in mHLA-DR expression has been associated with acquisition of secondary infection and mortality in small studies [13, 19, 20, 23, 24] although one study did not found an association [25]. The largest study to date included > 400 adult patients and found lower mHLA-DR values in non-survivors. However, the authors concluded that mHLA-DR expression was not suitable as predictive parameter due to limited discriminative ability [26].

In children, small studies have confirmed an association of reduced mHLA-DR with secondary infection or mortality in post-operative critically ill children [27], in children with critical illness for multiple reasons [28] and in septic children [16, 18]. In 30 paediatric septic patients, Manzoli et al. found an association of decreased mHLA-DR expression with mortality but not with secondary infections [18]. On our PICU, critically ill children are admitted with a wide range of infectious diseases including suspected community-acquired infections and hospital-acquired infections. More insight in mHLA-DR expression and the relation with mortality and secondary infections in these children can aid to identify patients who could benefit from immunostimulatory therapies.

Therefore, we performed a large study including children with a wide range of infectious diseases to assess the relation of mHLA-DR expression with adverse outcomes. The association of mHLA-DR expression at several timepoints and the change of mHLA-DR expression over time was studied and related to mortality and the acquisition of secondary infections.

## Methods

### Study design and population

This is a pre-planned prospective observational study embedded in the PERFORM project (Personalised Risk assessment in Febrile illness to optimise Real-Life Management across the European Union, www. perform2020.org) [29]. The overarching aim of PERFORM is to improve diagnosis and management of febrile children by development of a new diagnostic tests to discriminate bacterial from viral infections. For this particular study, we included critically ill children (aged 0–17 years) admitted to the level 3 PICU of Erasmus MC-Sophia Children’s hospital (Rotterdam, the Netherlands, mixed surgical/medical) between March 2017 and April 2019. Inclusion criteria included fever (body temperature of ≥ 38° C) and/or a suspected infection in patients who had an arterial or central venous line in situ. This comprised both children who were admitted through the Emergency Department (community-acquired infection) and children who were already admitted and developed an infection during hospital admission > 48 h after admission (hospital-acquired infection). For critically ill children, exclusion criteria were presence of chronic conditions that affect immune status (immunodeficiency, malignancy, chronic immunosuppressive medication including corticosteroids) and severe anaemia (haemoglobin < 6.5 g/dL). In addition, we included afebrile healthy controls who underwent elective surgery for a minor condition. For healthy controls, exclusion criteria included fever in the three week prior to surgery or presence of more than one chronic condition. To cover all ages, we aimed to include 20 healthy children in clinically relevant age groups: 0–1 years, 1–2 years, 2–5 years, 5–12 years and 12–16 years. Informed consent was obtained from children > 12 years and parents or legal guardians.

### Clinical data

Prospective clinical data were collected from electronic medical records. We collected baseline data on the first day of PICU admission or, in a case of suspected hospital-acquired infection, at the onset of the infectious disease episode on the PICU. Collected data included demographics, chronic conditions, Paediatric Risk of Mortality (PRISM) score [30], Paediatric Logistic Organ Dysfunction-2 (PELOD-2) probability of mortality [31] and signs of septic shock (sepsis and cardiovascular organ dysfunction as defined by Goldstein et al.) [32]. The cause of the initial infection was classified in proven/presumed bacterial, proven/presumed viral, unknown bacterial/viral or other, according to a published flowchart including clinical signs and symptoms and microbiological cultures/PCR [33]. In addition, for a period of 28 days following inclusion, we collected days of mechanical ventilation, days of inotropic support, survival status and number of PICU-free days at day 28.

### Outcome measures

The outcome measures included 28-day mortality and acquisition of a secondary infection defined by the surveillance definition of hospital-acquired infections by Centres for Disease Control and Prevention according to previous studies [7, 28, 34]. All suspected secondary infections during 28 days of follow-up after inclusion were reviewed by trained clinical researchers (NH, PK, JW) and discussed with one paediatric infectious disease specialist (CV). All researchers were blinded regarding mHLA-DR expression at the time of reviewing.

### Blood sampling and measurement of monocytic HLA-DR expression

Blood samples were taken either on PICU admission or at the onset of the infectious episode during PICU stay. Follow-up samples were taken at day 2–3, day 4–7 and once a week with a maximum of 5 blood draws in total. The number of time points in which follow-up sampling could be performed was restricted by logistic reasons, limitations of blood sample volumes and lack of parents’ consent. For healthy controls, a single sample was drawn during insertion of the peripheral catheter before start of surgery. Samples were analysed for mHLA-DR expression on a flowcytometer (FACSCanto-II, BectonDickinson) using the Anti-HLA-DR/Anti-Monocyte Quantibriteassay (BD Biosciences), as described previously [35, 36]. This assay approach uses an HLA-DR antibody conjugated to phycoerythrin (PE) in a 1:1 ratio as well as a mixture of beads to which a defined amount of PE molecules have been conjugated. In the end, this allows to calculate the number of HLA-DR-PE antibodies bound per cell (AB/c). Laboratory technicians conducting the assay were blinded for the clinical data.

### Data analysis

We compared results of mHLA-DR expression of patients with suspected infections vs healthy controls, and patients with and without secondary infections. Differences between the groups were tested for significance with Mann–Whitney *U* test, Student’s *t* test or Kruskal–Wallis test when appropriate. To assess the change of mHLA-DR expression over time during PICU admission, we calculated delta-mHLA-DR: the difference of mHLA-DR expression between the latest timepoint and the first timepoint.

In logistic regression analysis, we adjusted for age and baseline PELOD-2 score. We assessed the association of delta-mHLA-DR for acquisition of secondary infections, and for the composite outcome acquisition of secondary infections and/or 28-day mortality. Based on delta-mHLA-DR, patients were classified as improved (delta > 20% from baseline), declined (delta <  −20% from baseline) or stable (< 20% difference from baseline). The delta 20% from baseline was chosen to ensure sufficient numbers for statistical analysis. We tested the interaction between delta-mHLA-DR and delta-mHLA-DR group (improved, declined, stable) for secondary infection using the likelihood ratio test. In addition, we tested the interaction between delta-mHLA-DR and cause of the initial infection (proven/presumed bacterial, proven/presumed viral, unknown bacterial/viral or other). Next, we explored the association of delta-mHLA-DR in the subgroup with low mHLA-DR, defined as values below the 25^th^ centile at baseline in our population. As an exploratory analysis, we assessed the association of delta-mHLA-DR with PICU free days at day 28 adjusted for age and baseline PELOD-2 score. All data analyses were performed in R version 3.6 and a *p* value < 0.05 was considered significant.

## Results

### Study population

**Fig. 1:**
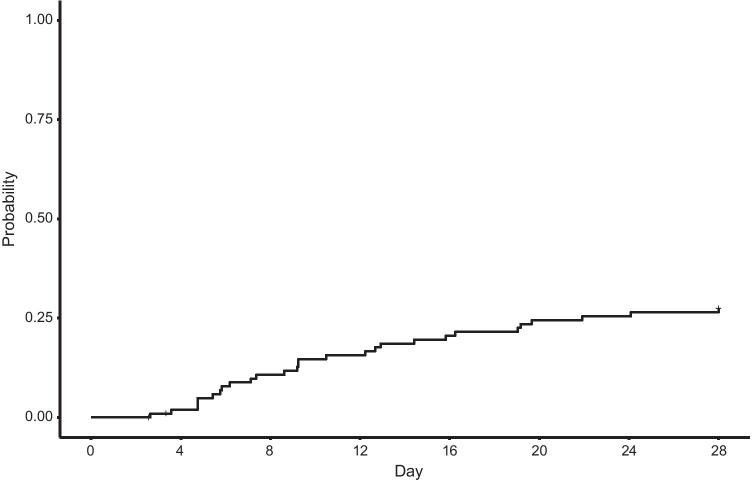
Cumulative incidence of secondary infection (*n* = 104)

We included 104 patients with suspected infection and 93 healthy controls with available mHLA-DR measurements. Compared to controls, infectious patients were younger (median 1.2 years [IQR 0.3–4.2] vs 3.6 years [IQR 1.1–9.9]) but were similar in sex ((male: 60% (62/104) vs 66% (61/93)) (additional file 1). Of the 104 infectious patients, the initial infection was a suspected community-acquired infection in 36% (*n* = 37) and a suspected hospital-acquired infection in 64% (*n* = 76). Details of the clinical syndromes are presented in additional file 2. Seven patients died (7%) and 28 patients (27%) acquired a secondary infection after inclusion which occurred after a median of 9 days (IQR 5–16 days) (Fig. 1, additional file 3). Compared to patients without a secondary infection, patients with a secondary infection were similar in age, sex, initial infection and baseline PELOD-2 and PRISM score. Patients with a secondary infection, however, had more ventilation and inotropes days and were admitted longer to the PICU (Table 1) than patients without a secondary infection. Comparing children with suspected community-acquired and hospital-acquired infections, no differences on baseline PRISM or PELOD-2 scores or occurrence of secondary infections were observed.

**Table 1:** Descriptive characteristics of critically ill children with suspected infections (*n* = 104)

	**Secondary infection**	**No secondary infection**	*p* value
	***n* = 28**	***n* = 76**	
**Age in years, median [IQR]**	1.3 (0.5–3.1)	1.0 (0.2–4.2)	0.47
**Male**	18 (64.3)	44 (57.9)	0.72
Any chronic underlying condition*	21 (75.0)	54 (71.1)	0.88
Pulmonary	5 (17.9)	11 (14.5)	
Prematurity	2 (7.1)	7 (9.2)	
Gastro-intestinal		3 (3.9)	
Neurological	2 (7.1)	7 (9.2)	
Cardio	15 (53.6)	36 (47.4)	
Endocrinologic	1 (3.6)		
Genetic	6 (21.4)	9 (11.8)	
**PRISM score at day 1, median [IQR]**	15 (12–22)	14 (9–19)	0.26
**PELOD-2 probability of mortality at day 1, median [IQR]**	2.2 (1.3–6.2)	1.4 (0.5–3.5)	0.11
**PICU admission duration in days during study period, median [IQR]**	19 (9–36)	8 (2–17)	0.003
**Days of invasive ventilation during study period**	7 (2–18)	2 (0–5)	0.003
**Days of inotropes during study period**	2 (0–9)	1 (0–2)	0.02
**Signs of septic shock at baseline**	9 (32.1)	15 (19.7)	0.26
**Phenotype initial infection**			0.08
Proven/presumed bacterial infections^a^	13 (46.4)	19 (25.0)	
Proven/presumed viral infections^b^	3 (10.7)	16 (21.1)	
Unknown bacterial/viral	3 (10.7)	6 (7.9)	
Other	9 (32.1)	35 (46.1)	
**Mortality**	4 (14.3)	2 (2.6)	0.07

*IQR*, interquartile range; *PELOD*-*2*, Paediatric Logistic Organ Dysfunction-2; *PRISM*, Paediatric Risk of Mortality

^*^Multiple categories possible for one patient

^a^*n* = 18 proven bacterial infections (*n* = 5 for secondary infection, *n* = 13 for no secondary infection)

^b^*n* = 2 proven viral infections (*n* = 0 for secondary infection, *n* = 2 for no secondary infection)

### Monocytic HLA-DR expression

**Fig. 2:**
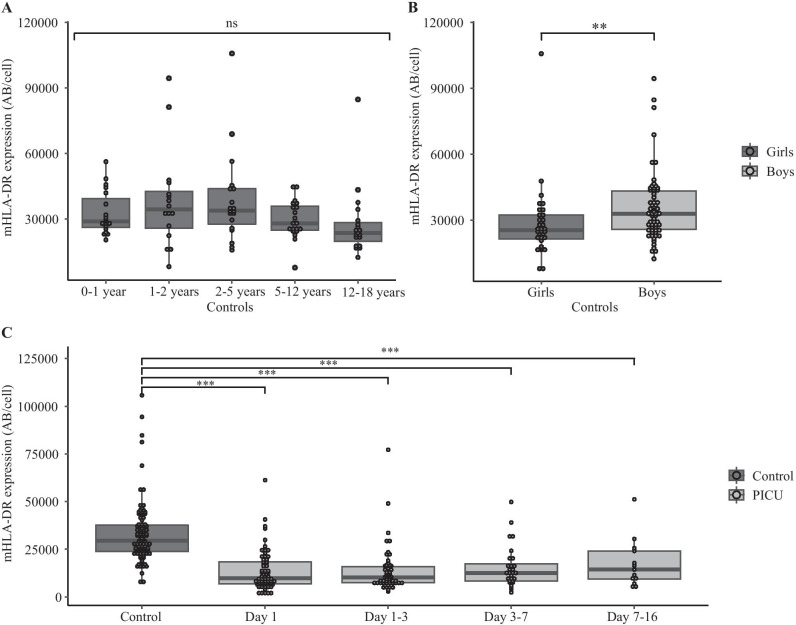
Monocyte HLA-DR expression. **A** mHLA-DR expression stratified for age group in controls. **B** mHLA-DR expression stratified for sex in controls. **C** mHLA-DR expression for controls and children with suspected infections at different timepoints. Ns, not significant; ***p < 0.001, **p < 0.01

In controls, mHLA-DR expression was 29,500 AB/cell (IQR 23,800–37,600) and was similar across age groups (0–1 years 28,000 [IQR 26,100–39300]; 1–2 years 34,300 [25700–42600]; 2–5 years 33,900 [27600–43800]; 5–12 years 28,000 [24900–35800]; 12–18 years 23,600 [19700–28300]) (Fig. 2a). In controls, boys had higher mHLA-DR expression than girls (32,900 AB/cell [IQR 25,700–43300] vs 25,400 [IQR 21,400–32200], *p* < 0.01) (Fig. 2b), but mHLA-DR expression in critically ill children was similar in girls and boys at all timepoints. In critically ill children, mHLA-DR expression was significantly lower than in controls at all timepoints (Fig. 2c). No differences were observed for baseline mHLA-DR expression in community-acquired infections or hospital-acquired infections.

### Association of monocytic HLA-DR expression with secondary infection

**Fig. 3:**
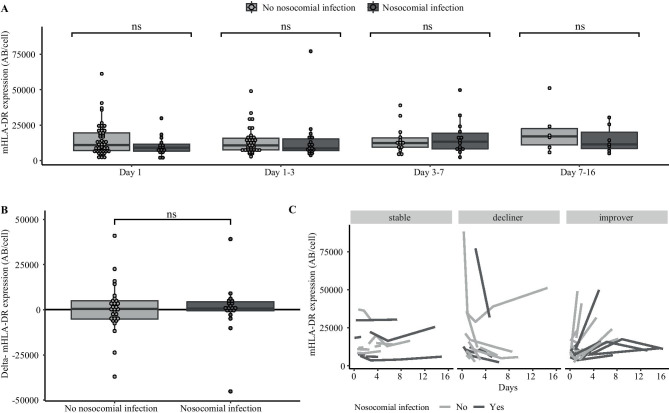
Secondary infection. **A** mHLA-DR expression stratified for secondary infections at different timepoints. **B** delta-mHLA-DR expression in patients with and without secondary infection. **C** mHLA-DR expression in secondary infection stratified for delta-mHLA-DR groups (stable, decliner, improver). Ns, not significant

mHLA-DR expression did not differ for patients with and without secondary infections at any of the timepoints (Fig. 3a). In patients with two or more measurements (*n* = 47), delta-mHLA-DR varied widely (range − 45,232 to 40,944 AB/cell). Delta-mHLA-DR expression did not differ for patients with and without secondary infection (712,000 AB/cell [IQR − 496 to 4371] vs (497,000 AB/cell [IQR − 5161 to 4896]) (Fig. 3b). Adjusted for age and PELOD-2 score, delta-mHLA-DR was not associated with acquisition of secondary infection (aOR 1.00 [95% CI 0.96–1.04]), or the composite outcome secondary infection/mortality (aOR 0.99 [0.96–1.04]). Occurrence of secondary infection did not differ for patients classified by delta-mHLA-DR as improved (8/19, 42%), declined (4/14, 29%) or stable (7/14, 50%), although numbers were small (Fig. 3c) (*p* > 0.05 for interaction). In addition, the association of delta-mHLA-DR did not vary for cause of infection groups (*p* > 0.05 for interaction). In the subgroup of patients with low mHLA-DR at baseline (< 7200 AB/cell, *n* = 26), delta-mHLA-DR was not associated with secondary infection (OR 1.0 [95% CI 0.7–1.6]). Lastly, adjusted for age and PELOD-2 score, delta-mHLA-DR was not associated with PICU free days at day 28 (*β* 0.05 [95% CI −0.15 to 0.26]).

## Discussion

### Main findings

In our study of critically ill children with suspected infections, mHLA-DR expression was significantly lower compared to healthy controls. No association was found between mHLA-DR expression and acquisition of secondary infections at any of the timepoints. In addition, change in mHLA-DR expression over time was not associated with the occurrence of secondary infections or the composite outcome secondary infection/mortality.

Our study also provides reference values of mHLA-DR expression in healthy children in different age groups and, similar to previous studies, we found no difference of mHLA-DR expression for age. Compared to one previous study in children using the same measurement method, we found similar mHLA-DR values in our large sample of controls [18]. Surprisingly, healthy boys had higher mHLA-DR expression compared to girls. A previous study including healthy children did not report any differences in sex for mHLA-DR expression [28]. This sex-difference in mHLA-DR was not observed in critically ill children, although their mHLA-DR levels were also influenced by disease severity. In two studies investigating immune markers in adults, pre-surgical mHLA-DR levels did not differ between men and women [37, 38]. As mHLA-DR expression did not differ in the various studied age groups, we did not perform an age-matched analysis.

Previous studies in children have investigated the relation of mHLA-DR expression with secondary infections in general ICU admissions or in septic shock: Boeddha et al. performed in our hospital, and Remy et al. reported a significant association, whereas Manzoli et al. did not [16, 18, 28]. Our study, using a larger cohort of children, did not show an association of mHLA-DR expression with secondary infection. Populations included in previous studies differed: they were based on small number of patients (range 30–37 patients) and included either solely septic patients or a general ICU population, whereas our study included children with suspected infections. We included both (suspected) community-acquired and hospital-acquired infections, to cover the wide range of suspected infections at the ICU. Although these two groups might have different immunological profiles or different risks for secondary infections, we did not observe differences in baseline mHLA-DR expression or occurrence of secondary infections. In our study, the proportion of patients who suffered from a secondary infection (28%) was higher than reported by other studies including general PICU populations (11–19%) [5–7].

Although it is widely established that mHLA-DR expression is a measure for immunosuppression, cut-off values for mHLA-DR defining immunosuppression in both adults and children are yet unclear. In different adult trials which investigated immunostimulatory therapies in septic patients, cut-offs of < 8000 mAB/cell and 10,000 AB/cell were used for patient selection [40; 41]. Tamulyte et al. found that different cut-off values for mHLA-DR expression (2000/5000/8000 molecules/cell) could not predict mortality, but the cut-offs of 2000 and 5000 could discriminate patients with a longer ICU stay and ventilation days. In their study, mHLA-DR measurement was performed using novel point-of-care flow cytometry. It is yet unclear whether these cut-off values can be extrapolated to children. Manzoli et al. suggested that a delta of < 1000 AB/cell was associated with mortality in septic children [18]. We hypothesized that different cut-offs may be needed for prediction of secondary infections. Therefore, we performed a subgroup analysis in the lowest mHLA-DR quartile but found no association of change in mHLA-DR expression with the occurrence of secondary infection.

Although we found that mHLA-DR expression is lower in our cohort of critically ill children compared to healthy controls, its clinical value is yet unclear. Our results show that in critically ill children with suspected infections, mHLA-DR expression has no additional value to identify patients who are at risk for secondary infection. The immunosuppressed state of critically ill children as measured by mHLA-DR was not related to acquisition of secondary infections or with duration of ICU stay.

Previous studies have used immunostimulating therapies as granulocyte–macrophage colony-stimulation factor (GM-CSF) to restore mHLA-DR levels and improved patient outcomes [39–43]. In addition, interferon-gamma improved the immune response in a child suffering from refractory candidemia with extremely low mHLA-DR [44]. Currently, these immunostimulating therapies are not routinely used in clinical practice due to the lack of predictive ability on the individual level [26, 45]. Hence, future trials in children should focus on accurate identification of immunosuppression using point-of-care devices in the assessment of mHLA-DR and prediction could be improved by using multiple biomarkers involved in other pathways such as programmed death (PD)-1 [46].

The main strengths of this study include its prospective design and inclusion of large number of critically ill children with a wide variety of infections. Second, we collected detailed clinical data with complete follow-up on all patients. In addition, we used a standardized method for mHLA-DR measurement to facilitate comparison with other studies [35, 36]. The major limitation of this study is that we were not able to collect follow-up samples in all patients: patients were either discharged from the PICU, had their central line removed, no consent was given for multiple blood sampling, blood volume restrictions were reached or mHLA-DR measurement was not possible due to logistic reasons. It is possible that follow-up samples were collected in critically ill children with more severe disease, which could limit the generalizability of our results to the general PICU population. Furthermore, secondary infections may occur more frequent in patients who have more ventilation days, more ICU days or invasive medical devices such as urinary catheters and central venous catheters. Although we adjusted for PELOD-2 in our analysis, we did not have enough power to include all these confounders in our analysis. Lastly, some patients in our cohort had surgery during the follow-up period which could have influenced mHLA-DR levels [27].

## Conclusion

In this single-centre study of mHLA-DR expression in children admitted to PICU with suspected infections, we confirm that critically ill children have lower mHLA-DR expression than controls. Decreased mHLA-DR and change in mHLA-DR was not associated with the acquisition of secondary infections. Therefore, in this population, mHLA-DR expression is not valuable for identifying children at risk for secondary infections.

## Supplementary Information

Below is the link to the electronic supplementary material.

Supplementary file1 (DOCX 16 KB)

## Data Availability

A data set containing individual participant data will be made available in a public data repository containing a specific DOI upon publication. The data will be anonymized and will not contain any identifiable data.

## Abbreviations

GM-CSF: Granulocyte-macrophage colony-stimulation factor
mHLA-DR: Human leukocyte antigen-DR expression on monocyt
PERFORM: Personalised Risk assessment in Febrile illness to optimise Real-Life Management across the European Union
PICU: Paediatric intensive care unit
PRISM: Paediatric risk of Mortality
PELOD-2: Paediatric Logistic Organ Dysfunction-2
