# Supplementary material for: Association of monocyte HLA-DR expression over time with secondary infection in critically ill children: a prospective observational study
Source: Eur J Pediatr. 2021 Nov 10;181(3):1133–42. doi: 10.1007/s00431-021-04313-7 (PMC8897323; doi:10.1007/s00431-021-04313-7)
Supplement: Supplementary file 1 — Supplementary file1 (DOCX 16 KB) [file 431_2021_4313_MOESM1_ESM.docx]

## Additional file 1: characteristics of critically ill children with suspected infections and controls

|  | **patients (n=104)** | **Control (n=93)** | P-value |
| --- | --- | --- | --- |
| Age in years, median [IQR] | 1.1 [0.2-4.1] | 3.6 [1.1-9.9] | <0.01 |
| 0-1 year | 51 (49.0) | 19 (20.4) |  |
| 1-2 years | 13 (12.5) | 16 (17.2) |  |
| 2-5 years | 16 (15.4) | 19 (20.4) |  |
| 5-12 years | 10 (9.6) | 20 (21.5) |  |
| 12-18 years | 14 (13.5) | 19 (20.4) |  |
| Male | 62 (59.6) | 61 (65.6) | 0.6 |
| Type of surgery |  |  |  |
| Urology |  | 49 (52.7) |  |
| Plastic surgery |  | 18 (19.4) |  |
| Orthopedic surgery |  | 18 (19.4) |  |
| Other |  | 8 (8.6) |  |

mHLA-DR levels in controls and per age group

|  | mHLA-DR AB/cell (/1000) |
| --- | --- |
|  | Median (IQR) |
| All controls, n=93 | 29.5 (23.8-37.6) |
| 0-1 year, n=19 | 28.9 (26.1-39.3) |
| 1-2 years, n=16 | 34.3 (25.7-42.6) |
| 2-5 years, n=19 | 33.9 (27.6-43.8) |
| 5-12 years, n=20 | 28.0 (24.9-35.8) |
| 12-18 years, n=19 | 23.6 (19.7-28.3) |

mHLA-DR levels in PICU patients

|  | mHLA-DR AB/cell (/1000) |
| --- | --- |
|  | Median (IQR) |
| Day 1 (n=66) | 9.7 (6.9-18.3) |
| Day 1-3 (n=55) | 10.2 (7.5-15.9) |
| Day 4-7 (n=28) | 12.5 (8.4-17.4) |
| Day 8-16 (n=13) | 14.4 (9.3-24.1) |

## Additional file 2: clinical syndrome of initial infection

| **Clinical syndrome of initial infection (n=104)** | **N (%)** |
| --- | --- |
| Lower respiratory tract infection | 35 (33.7) |
| Upper respiratory tract infection | 4 (3.9) |
| Central nervous system infection | 5 (4.8) |
| Gastro-intestinal tract infection / surgical abdomen | 5 (4.8) |
| Sepsis | 6 (5.8) |
| Undifferentiated fever | 20 (19.2) |
| Central line-associated bloodstream infection | 7 (6.7) |
| Post-operative fever | 18 (17.3) |
| Cardiac | 2 (1.9) |
| Other | 2 (1.9) |

## Additional file 3: details of secondary infections

|  | **Secondary infections** |
| --- | --- |
|  | n=28 |
|  | **n (%)** |
| Blood stream infection/clinical sepsis | 7 (25) |
| Gastrointestinal system | 4 (14.3) |
| Other lower respiratory tract | 1 (3.6) |
| Pneumonia | 4 (14.3) |
| Surgical site | 5 (17.9) |
| Skin and soft tissue | 1 (3.6) |
| Urinary tract | 5 (17.9) |
| Eye, ear nose, throat or mouth infection | 1 (3.6) |
